# Implications of obesity and insulin resistance for the treatment of oestrogen receptor-positive breast cancer

**DOI:** 10.1038/s41416-024-02833-1

**Published:** 2024-09-09

**Authors:** Sohail Rooman Javed, Aglaia Skolariki, Mohammed Zeeshan Zameer, Simon R. Lord

**Affiliations:** Department of Oncology, University of Oxford, Oxford, UK

**Keywords:** Breast cancer, Breast cancer

## Abstract

Breast cancer is the most common cancer in women, and incidence rates are rising, it is thought in part, due to increasing levels of obesity. Endocrine therapy (ET) remains the cornerstone of systemic therapy for early and advanced oestrogen receptor-positive (ER + ) breast cancer, but despite treatment advances, it is becoming more evident that obesity and insulin resistance are associated with worse outcomes. Here, we describe the current understanding of the relationship between both obesity and diabetes and the prevalence and outcomes for ER+ breast cancer. We also discuss the mechanisms associated with resistance to ET and the relationship to treatment toxicity.

## Introduction

The obesity epidemic is contributing to the rising incidence rates of breast cancer, which remains the most common cancer for women worldwide [1]. Furthermore, the relationship between breast cancer and metabolic disorders, specifically obesity and insulin resistance, increases the complexity of breast cancer treatment, posing unique challenges in managing toxicities and treatment resistance.

As described by the World Health Organisation (WHO), a body mass index (BMI) of 30 kg/m^2^ or greater is considered obese and is present in 13% of the world’s adult population, with greater prevalence in the Western world [2]. Obese patients with ER+ breast cancer are at a greater risk of cancer development, recurrence, and mortality [3–6], even after accounting for confounding variables such as concomitant diseases and chemotherapy underdosing [7]. Obesity also increases the risk of insulin resistance, characterised by cellular insensitivity to insulin, and is associated with a cluster of conditions, including hypertension, hyperglycaemia, central adiposity, and dyslipidaemia, known as metabolic syndrome [2]. These variables collectively raise the risk of developing Type 2 diabetes mellitus (T2DM), which in itself is associated with increased cancer risk [8].

In addition, there is growing evidence that the metabolic abnormalities associated with obesity and insulin resistance may have a detrimental impact on the efficacy of ET [4, 9, 10]. In this article, several hypotheses for this impaired efficacy have been explored, including the impaired regulation of aromatase in obesity as well as the role of PI3K, leptin, and FGFR1 signalling. Obesity and insulin resistance may also impact the altered toxicity profile of systemic cancer treatments.

In this paper, we consolidate the current understanding of the impact of obesity and diabetes on cancer risk, treatment outcomes, and toxicity in ER+ breast cancer.

## Obesity, diabetes, breast cancer risk and outcome

In the last four decades, the rising incidence of breast cancer has been partly attributed to the introduction of national screening programmes worldwide, promoting the identification of small, early-stage tumours with favourable prognoses [11]. However, multiple epidemiological studies across diverse ethnic populations presented in Table 1, have associated the simultaneous rise in obesity levels with the increasing breast cancer incidence.

**Table 1:** Epidemiological studies investigating a relationship between obesity and risk of postmenopausal breast cancer.

Study title	Key findings	Sample size	Country
Obesity and breast cancer risk in Korean women [151]	Postmenopausal women: Increased breast cancer risk with higher BMI. Risk compared to BMI 18.5–23 kg/m^2^: <18.5: aHR 0.82 (0.75–0.89) 23–25: aHR 1.11 (1.08–1.14) 25–30: aHR 1.28 (1.25–1.32) ≥30: aHR 1.54 (1.47–1.62) Premenopausal women: Decreased breast cancer risk with higher BMI. Risk compared to BMI 18.5–23 kg/m^2^: <18.5: aHR 1.02 (0.94–1.11) 23–25: aHR 1.01 (0.97–1.05) 25–30: aHR 0.95 (0.91–0.98) ≥30: aHR 0.90 (0.82–0.98)	6,467,388 (57,626 breast cancer cases)	South Korea
Body mass index and breast cancer: analysis of a nationwide population-based prospective cohort study on 1,393,985 Taiwanese women [152]	Postmenopausal women: Adjusted hazard ratio (95% CI) = 0.78 (0.63, 0.96), 1.19 (1.12, 1.27), 1.31 (1.21, 1.41), 1.53 (1.38, 1.71) and 1.65 (1.27, 2.13) for BMI < 18.5, 24–26.9, 27–29.9, 30–34.9 and ⩾35, respectively Premenopausal women: Adjusted hazard ratio (95% CI) = 0.94 (0.81, 1.1), 0.98 (0.91, 1.04), 1.02 (0.93, 1.13), 1.01 (0.86, 1.18) and 0.82 (0.54, 1.24) for BMI < 18.5, 24–26.9, 27–29.9, 30–34.9 and ⩾35, respectively	1,393,985 (14,008 breast cancer cases)	Taiwan
Cancer incidence and mortality in relation to body mass index in the Million Women Study: cohort study [153]	Relative risk of breast cancer incidence according to BMI (95% CI) BMI 25–27.4 :1.10 (1.04–1.16) BMI 27.5–29.5: 1.21 (1.13–1.29) BMI > 30: 1.29 (1.22–1.36)	1.2 million (6808 breast cancer cases)	United Kingdom
Body size in early life and the risk of postmenopausal breast cancer [154]	Greater BMI at age 60 was associated with an increased risk of postmenopausal breast cancer (RR per 5 kg/m^2^ = 1.20, 95% CI 1.18–1.22)	342,079 (15,506 breast cancer cases)	United Kingdom
Body size and breast cancer risk (EPIC) [155]	Obesity linked to increased breast cancer risk in postmenopausal women not receiving hormone replacement therapy (HRT); inverse relationship in HRT users: 31% excess risk compared to women with BMI < 25 over 4.7-year follow-up.	176,886, (1879 breast cancer cases)	9 European countries
Excess body weight, weight gain and obesity-related cancer risk in women in Norway: the Norwegian Women and Cancer study [156]	Excess body weight raised the risk of postmenopausal breast cancer, with overweight (BMI 25– < 30 kg/m^2^) showing an HR of 1.13 (95% CI: 1.00–1.27) and obesity (BMI ≥ 30 kg/m^2^) showing a borderline significant HR of 1.20 (95% CI: 1.00–1.44, *P* = 0.05).	138,746 (3836 breast cancer cases)	Norway
Adiposity, Adult Weight Change, and Postmenopausal Breast Cancer Risk [157]	Weight gain from age 18 to the current age linked to higher breast cancer risk in hormone therapy non-users (RR 2.15, 95% CI: 1.35–3.42 for a ≥ 50-kg gain vs stable weight). The risk from adult weight change was stronger in women with later menarche (RR 4.20, 95% CI: 2.05–8.64 for ≥15 years) compared to earlier menarche (RR 1.51, 95% CI: 1.11–2.06 for ages 11–12; *P* = 0.007 for interaction).	99,039 (2111 breast cancer cases)	United States
Obesity, body size, and breast cancer risk (Women’s Health Initiative) [158]	Higher BMI in non-HRT postmenopausal women associated with increased breast cancer risk, relative risk 2.52; 95% confidence interval (CI) = 1.62–3.93	85,917 (1030 breast cancer cases)	United States
Body weight and breast cancer risk among Swedish women [159]	Positive association between obesity and ER + PR+ tumour risk compared to normal weight: RR = 1.67 (1.34–2.07) Inverse association between obesity and all PR- tumour risk: RR = 0.68 (0.47–0.98) Significant heterogeneity between ER + PR+ and PR- tumour risks: *P* (heterogeneity) <0.0001 Stronger association of obesity with ER + PR+ tumours in: never-users of HRT: RR = 1.90 (1.38–2.61) Those without a family history of breast cancer: RR = 1.82 (1.45–2.29)	51,823 (1188 breast cancer cases)	Sweden
Overall and central adiposity and breast cancer risk (Sister Study) [160]	Positive association between weight, BMI, waist circumference, waist-to-hip ratio, and breast cancer risk, with hazard ratios being greater in postmenopausal women. A non-linear increase in overall breast cancer risk was observed for increased categories of BMI. Estimates were stronger and monotonic for women with ER + PR+ invasive tumours (25–29 kg/m^2^, HR = 1.45, 95% CI 1.23, 1.71; 30–34.9 kg/m^2^, HR = 1.42, 95% CI 1.16, 1.75; ≥35 kg/m^2^, HR = 1.49, 95% CI 1.18, 1.88, vs. 18.5–24.9 kg/m^2^).	50,884 (2009 breast cancer cases)	United States
Obesity/weight gain and breast cancer risk: findings from the Japan collaborative cohort study for the evaluation of cancer risk [161]	Among postmenopausal women, adjusted HR increased with BMI. Risk increased among women with a BMI of 24 or higher HR: 1.50 (95% CI: 1.09–2.08) for BMI of 24–28.9, and 2.13 (1.09–4.16) for BMI ≥ 29) compared with women with a BMI of 20–23.9.	36,164 (234 breast cancer cases)	Japan
Maximum and Time-Dependent Body Mass Index and Breast Cancer Incidence Among Postmenopausal Women in the Black Women’s Health Study [162]	For overall breast cancer, the HR for BMI ≥ 35 versus BMI < 25 was 1.24 (95% CI: 1.02, 1.50). Stronger association for ER+ breast cancer (HR = 1.42, 95% CI: 1.10, 1.84)	31,028 (1384 breast cancer cases)	African American

A primary search for publications in the PubMed database from January 2000 to January 2024 for epidemiological studies investigating the relationship between obesity and risk of postmenopausal breast cancer was achieved using certain text keywords: breast cancer, obesity, body weight, BMI and epidemiology. An additional literature search was carried out with no systematic component. We limited the studies to those that included greater than 30,000 patients.

As body size and fat mass increase, endogenous oestrogen production is heightened, while sex hormone-binding globulin levels decrease. This hormonal imbalance is hypothesised to account for the link between obesity and an elevated risk of breast cancer in postmenopausal women [12, 13].

Although this link is well established in postmenopausal women, where increased androgen aromatisation in adipose tissue leads to higher oestrogen levels [14–16], the relationship between obesity and breast cancer risk in premenopausal women remains less clear. In this population, obesity is associated with a reduced incidence of breast cancer [14, 17]. For example, a large prospective multicentre analysis of over 700,000 premenopausal women showed that higher BMI during early adulthood is associated with a reduced risk of developing future breast cancer. This inverse association was stronger at younger ages and persisted across all BMI distributions, suggesting that increased adiposity early in life might have a protective effect against premenopausal breast cancer [18]. To explain this paradoxical risk reduction, it has been proposed that there is decreased oestrogen exposure in premenopausal obese women due to increased anovulatory menstrual cycles, a later decline in progesterone levels during menstruation, and longer menstruation [14–16, 19]. However, a longitudinal study did not find a clear relationship between BMI and ovulation-related variables like probable polycystic ovarian syndrome, oral contraceptives, and infertility secondary to an ovulatory disorder, which has cast doubt on this view [14, 18]. There is added complexity when considering findings from a pooled analysis of seven prospective studies, which investigated how circulating oestrogen and androgens affect premenopausal breast cancer risk. This study indicated that although total oestradiol levels were inversely related to BMI and positively associated with cancer risk, suggesting that the lower risk in obese women might stem from its reduced levels, free oestradiol, oestrone, and androgens such as DHEAS, testosterone, and free testosterone were found to be positively associated both with BMI and premenopausal breast cancer risk [20].

Aside from decreased oestrogen exposure, a net reduction in progesterone production is also hypothesised to account for decreased breast cancer risk in pre-menopause [21]. Progesterone is considered a major mitogen in the adult mammary epithelium in both mice and humans and has been linked to mammary carcinogenesis [22]. In the obese, premenopausal population, increased total oestrogen levels from adipose tissue and ovarian oestrogen production lead to enhanced negative feedback on hypothalamic pituitary-controlled gonadotropin release, therefore reducing ovarian steroid synthesis and progesterone production. Unlike postmenopausal women who produce no ovarian oestrogen [21], the oestrogen-progesterone imbalance in premenopausal obese women has been put forward as an explanation for the reduced breast cancer risk observed in this group.

In postmenopausal women, obesity has also been shown to increase breast cancer-related disease recurrence and mortality [7, 9, 23]. A meta-analysis of 82 follow-up studies demonstrated that breast cancer survivors with a higher BMI have worse overall and breast cancer-specific survival [7]. Overweight or obese breast cancer patients often present with larger tumours, higher-grade malignancy, and more positive lymph nodes at diagnosis. However, even after adjusting for these known prognostic factors, obesity independently raises the risk of distant metastases and breast cancer-related death [24].

Similarly, several meta-analyses in the last 20 years, as detailed in Table 2, have consistently shown that diabetes is associated with an increased incidence of breast cancer. It has been proposed that diabetes contributes to the onset of breast cancer via various mechanisms, such as mitogenic hyperinsulinaemia/insulin-like growth factor (IGF) pathway signalling, hyperglycaemia, inflammation caused by excess fat, and alterations in the levels of sex hormones [25]. These mechanisms are discussed further later in this article.

**Table 2:** Meta-analyses of diabetes and breast cancer risk.

Study title	Year	Key findings
Diabetes and incidence of breast cancer and its molecular subtypes: a systematic review and meta-analysis [163]	2024	70 studies: 24 case–control; 46 cohort. Diabetes was associated with an overall increased risk of breast cancer (RR = 1.20, 95% CI: 1.11–1.29). Postmenopausal women had an elevated risk of developing breast cancer (RR = 1.12, 95% CI: 1.07–1.17). No association between diabetes and breast cancer risk among premenopausal women (RR = 0.95, 95% CI: 0.85–1.05). Diabetes associated with significantly increased risk of (ER)+ breast cancer (RR = 1.09, 95% CI: 1.00–1.20), ER- (RR = 1.16, 95% CI: 1.04–1.30).
Breast cancer risk for women with diabetes and the impact of metformin: a meta-analysis [164]	2022	30 studies, 821,527 cases of breast cancer: In type 2 diabetic females, breast cancer RR = 1.15 (95% CI, 1.09–1.21). Adjusted for BMI: RR = 1.22 (95% CI, 1.15–1.30). Adjusted for BMI & menopause: RR = 1.20 (95% CI, 1.05–1.36). Metformin users vs. non-users, breast cancer RR = 0.82 (95% CI, 0.60–1.12).
Diabetes increases the risk of breast cancer: a meta-analysis [165]	2012	43 studies, 422,631 cases: 40 on women’s breast cancer, 6 on men’s and women’s breast cancer. Women with diabetes had increased breast cancer risk (OR 1.20, 95% CI 1.13–1.29). Type 2 diabetes: unchanged association (OR 1.22, 95% CI 1.07–1.40). Gestational diabetes: no association (OR 1.06, 95% CI 0.79–1.40). Insufficient data for type 1 diabetes. Males with diabetes showed increased breast cancer risk, not statistically significant (OR 1.29, 95% CI 0.99–1.67).
Diabetes and breast cancer risk: a meta-analysis [166]	2012	Studies included a total of 56,111 breast cancer cases. 39 studies: breast cancer SRR in diabetic women = 1.27 (95% CI, 1.16–1.39). Prospective studies: SRR = 1.23 (95% CI, 1.12–1.35). Retrospective studies: SRR = 1.36 (95% CI, 1.13–1.63). Type 1 diabetes/premenopausal: no association (SRR = 1.00 (95% CI, 0.74–1.35) and 0.86 (95% CI, 0.66–1.12), respectively). BMI-adjusted: SRR = 1.16 (95% CI, 1.08–1.24). Non-BMI-adjusted: SRR = 1.33 (95% CI, 1.18–1.51).
Association between diabetes mellitus and breast cancer risk: a meta-analysis of the literature [167]	2011	16 studies (2000–2010). A total of 730,069 patients. Diabetes linked to 23% increased breast cancer risk, notably in postmenopausal women (RR = 1.25, 95% CI 1.20–1.29). Significant correlation in Europe (RR = 1.88, 95% CI 1.56–2.25), and America (RR = 1.16, 95% CI 1.12–1.20), not significant in Asia (RR = 1.01, 95% CI 0.84–1.21). Diabetes raised breast cancer mortality (RR = 1.44, 95% CI 1.31–1.58).
Diabetes mellitus and risk of breast cancer: a meta-analysis [168]	2007	20 studies (5 case–control and 15 cohort studies). Diabetes associated with increased breast cancer risk (RR = 1.2, 95% CI 1.12–1.28).

A primary search for publications in the PubMed database from January 2005 to January 2024 for meta-analyses investigating the relationship between diabetes and risk of postmenopausal breast cancer was achieved using the text keywords: breast cancer, diabetes and meta-analysis.

Furthermore, there is an approximate 40% increase in mortality following a breast cancer diagnosis among postmenopausal women with diabetes compared to women without diabetes. Nevertheless, this increase may, at least in part, be due to diabetes-related comorbidities [26]. Breast cancer-specific mortality also appears to be higher in diabetic women, although it is uncertain if mortality worsens with increasing severity of type 2 diabetes [27].

## Genetic links and shared susceptibility in obesity, diabetes, and breast cancer

Various hypotheses have been proposed to explain the frequent co-occurrence of obesity, diabetes and breast cancer, with one of the most prominent being the shared genetic aetiology. Recent advancements in genetic research, particularly through large Genome-Wide Association Studies (GWAS), have revealed that specific genetic variants are associated with these complex diseases across different populations.

Several variants that are associated with T2DM have also been linked to breast cancer. Notable examples include polymorphisms mapping to loci at 10q25.2 and 9p21.3 at which transcription factor 7-like 2 (TCF7L2) and cyclin-dependent kinase inhibitor 2A/B (CDKN2A/B) have been proposed as the target genes and both of which are involved in signalling pathways that regulate cell-cycle progression and proliferation [28–31]. The first obesity susceptibility locus discovered by GWAS mapped to 16q12.2, proximal to the fat mass and obesity-associated (FTO) gene which has been recognised as a regulator in DNA repair mechanisms, DNA damage and inflammatory responses. Polymorphisms at this locus have also been associated with breast cancer risk [32–36]. Furthermore, the FTO-encoded protein, an RNA N6-methyladenosine (m6A) demethylase, has been implicated in breast tumourigenesis and progression [37, 38].

Recently, interest in the FTO gene has been renewed following a systematic analysis exploring the potential overlap of known GWAS risk variants for obesity, T2DM and breast cancer. This study identified 91 candidate variants in linkage disequilibrium using datasets from the 1000-Genomes Project to analyse candidate haplotypic blocks. Surprisingly, all variants were located within the vicinity of the FTO gene, thus highlighting the significant association of this locus with these diseases and strengthening the hypothesis of a shared genetic basis [39].

However, conflicting evidence from previous case–control studies in women of various ethnicities has questioned the potential pleiotropic effects of these risk variants on breast cancer, obesity and diabetes traits [40–42]. In addition, a case–control study involving U.S. Caucasian women found non-significant correlations between intronic and intergenic single nucleotide polymorphisms (SNPs) located in or near 29 diabetes-related genes and breast cancer incidence and mortality, casting further uncertainty on the functional significance of these variants in relation to breast cancer risk [43]. Finally, Mendelian randomisation analyses have been implemented to infer causality between genetic instruments associated with obesity and diabetes and breast cancer risk [44–46]. This method provides clearer insights into causal associations by reducing bias and confounding, as well as mitigating reverse causation. Nevertheless, further mechanistic studies will be required to elucidate the underlying biological pathways and interactions that drive these associations.

### Mechanisms of obesity-induced carcinogenesis and treatment resistance in breast cancer

**Fig. 1:**
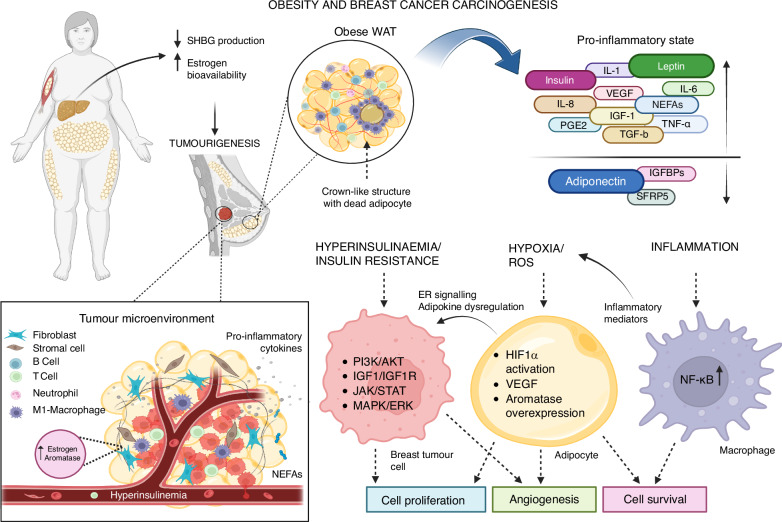
Summary of mechanisms of breast cancer carcinogenesis in obesity. In the presence of obesity, decreased SHBG production and heightened aromatase expression in the breast adipose stromal cells lead to increased oestrogen bioavailability and biosynthesis, a major contributor to the development and progression of ER+ breast cancer in postmenopausal women. The expanded adipose tissue is characterised by dysfunctional adipocytes and crown-like structures, and immune cell infiltration that accentuates a pro-inflammatory state. The state of hyperinsulinaemia and hyperglycaemia, concurrent with the release of adipokines, notably leptin and pro-inflammatory mediators by the macrophages contribute to the activation of pro-tumourigenic and metabolic pathways. Adipocyte hypoxia stabilises HIF-1α and VEGF upregulation, promoting angiogenesis. Together, these factors activate signalling cascades such as PI3K/AKT and MAPK/ERK, driving cell proliferation, survival, and breast cancer progression. SHBG sex hormone-binding globulin, WAT white adipose tissue, IL-1 interleukin 1, IL-6 interleukin 6, IL-8 interleukin 8, VEGF vascular endothelial growth factor, NEFAs non-esterified fatty acids, PGE2 prostaglandin E2, IGF-1 insulin-like growth factor 1, TGF-β transforming growth factor beta, TNF-α tumour necrosis factor alpha, IGFBPs insulin-like growth factor binding proteins, SFRP5 secreted frizzled-related protein 5, ER oestrogen receptor, ROS reactive oxygen species, HIF-1α hypoxia-inducible factor 1-alpha, NF-κB nuclear factor kappa B, PI3K/AKT phosphoinositide 3-kinase/protein kinase B, IGF-1R insulin-like growth factor 1 receptor, JAK/STAT Janus kinase/signal transducer and activator of transcription, MAPK/ERK mitogen-activated protein kinase/extracellular signal-regulated kinase. (Created with BioRender.com.).

Breast cancer development in the context of obesity has been linked to increased inflammation in adipose tissue, marked by macrophage infiltration and the formation of crown-like structures (CLS) around dead adipocytes. This increased inflammation within the adipose tissue microenvironment has been shown in obese mouse models and is associated with increased cell proliferation and higher levels of inflammatory cytokines, including TNF-alpha, IL-1β and Cox-2, as well as insulin resistance [47–49]. In a study of women undergoing mastectomy or breast cancer surgery, CLS was detected in 40% of cases, and was associated with higher levels of insulin, glucose, leptin, triglycerides, C-reactive protein and IL-6 [50]. The presence of CLS in breast tissue is linked to an increased risk of breast cancer and a poorer prognosis, with evidence showing associations with metastasis and decreased overall survival [49, 51, 52]. Several mechanisms may contribute to CLS formation and resistance to ET. Understanding and selectively targeting these mechanisms could affect both breast cancer development and associated insulin resistance. A summary of the mechanisms of breast cancer carcinogenesis in obesity is described in Fig. 1.

#### RANKL/TNF-alpha/NF-κB activation

The accumulation of macrophages in CLS is thought to be caused by a decrease in macrophage apoptosis within obese adipose tissue through the activation of the transcription factor NF-κB [53]. NF-κB has been found to be activated in human breast cancer cell lines and is considered critical in the genesis of ET resistance in ER+ breast cancer, as it has been shown to promote tamoxifen resistance, early recurrence, metastasis, and worse overall survival [53–56]. There also appears to be cross-talk between ER and NF-κB, potentially working in tandem to support breast cancer cell survival and transition to a more aggressive phenotype [57]. The upregulation of NF-κB is independently associated with hyperinsulinemia, and reduced β-cell function [58].

Novel therapies inhibiting NF-κB gene activation could therefore potentially prevent ER+ tumour recurrence and restore endocrine responsiveness. Preclinical studies indicate that suppressing NF-κB significantly enhances the sensitivity of resistant breast cancer tumour cells to tamoxifen [59, 60]. Riggins et al. demonstrated that pharmacologic inhibition of NF-κB by parthenolide, a small molecule inhibitor against NF-κB, could restore fulvestrant-mediated suppression of growth in breast cancer cell lines [61]. Despite this preclinical data, clinical trials exploring NF-κB inhibition have not been promising to date. Three Phase II studies investigating bortezomib, a proteosome inhibitor that blocks the NF-κB pathway, as a single agent or in combination with ET, did not elicit an objective tumour response in metastatic breast cancer patients [62–64].

Targeting upstream or downstream signals of NF-κB may provide more promising therapeutic prospects. RANK ligand (RANKL), a TNF-related molecule, has been shown to activate NF-κB in preclinical studies and thereby promote proliferative changes in the mammary epithelium as well as epithelial-mesenchymal transition, which induces tumour cell migration, invasion and metastasis [65–67]. Systemic and hepatic blockage of RANKL signalling can also improve hepatic insulin sensitivity and glucose tolerance [58]. Currently in clinical practice, the use of the RANKL inhibitor denosumab is not extended beyond the prevention or treatment of osteoporosis, primarily due to disappointment on its efficacy in improving disease-free survival (DFS) in patients [68, 69]. However, the potential of RANKL inhibitors, particularly denosumab, to counteract NF-κB-mediated resistance in ER+ breast cancer may still merit further clinical investigation.

Similarly, TNF-alpha, a cytokine acting upstream of NF-κB, has been shown to induce proliferation in murine mammary tumour cells [70]. By upregulating PTEN and suppressing the AKT/eNOS/NO signalling pathway, TNF-alpha also contributes to vascular insulin resistance [71]. Infliximab, which binds to an neutralises TNF-alpha, was found to be tolerable in patients with advanced cancer with some evidence of on-target activity [72]. In metastatic breast cancer, a Phase II clinical trial demonstrated the safety of another anti-TNF-alpha agent, etanercept, in heavily pre-treated patients, although more research is required to understand efficacy and any treatment role [73]. TNF-alpha blockade may also have a role in overcoming resistance to anti-PD-1 therapy, and combination therapy should be assessed for feasibility [74].

#### Hypoxia and induction of hypoxia-inducible factor 1-alpha (HIF-1α)

The activation of NF-κB in obesity-related breast cancer may be driven by adipocyte hypoxia [75]. It is hypothesised that adipocyte hypertrophy without hyperplasia leads to accelerated tissue growth with insufficient supportive angiogenesis [76]. Hypoxia in turn triggers the activation of hypoxia-inducible factors (HIFs), which are associated with increased proliferation and expression of ER and VEGF, suggesting a possible relationship with more aggressive tumours [77]. HIF-1α expression is associated with poorly differentiated breast cancer, a higher pathological stage, and poor treatment response and outcome [77, 78]. Obesity is also associated with elevated HIF-1α mRNA and protein in adipose tissue [79], while HIF-1α activation in macrophages is associated with the development of insulin resistance and glucose metabolism in addition to pro-tumour mechanisms [80]. This may be in part due to HIF-1α-induced upregulation of insulin receptor substrate 2 (IRS-2), which is an important mediator of insulin, glucose metabolism, and mitogenesis [81]. PI3K and downstream signalling effectors AKT and mTOR are activated through the recruitment of the IRS proteins.

Hypoxia is also a recognised driver of ET resistance, with elevated expression of HIF-2α observed in endocrine-resistant ERα-positive breast cancer cell lines [78, 82]. Introducing HIF-2 into previously sensitive cells leads to their development of resistance to antioestrogens and inhibiting HIF-2α signalling can restore sensitivity in cells that have become resistant to ET [82]. Additionally, established HIF inhibitors such as digoxin and acriflavine appear to have activity against breast cancer metastatic niche formation [83], and have been shown to decelerate diet-induced obesity by various mechanisms in mouse studies, including decreasing lipogenesis [84–86]. Therefore, focusing on HIF inhibitors may not only help overcome resistance to ET in obesity but also provide valuable insights into preventing diet-induced obesity.

#### PI3K–AKT–mTOR pathway activation

**Fig. 2:**
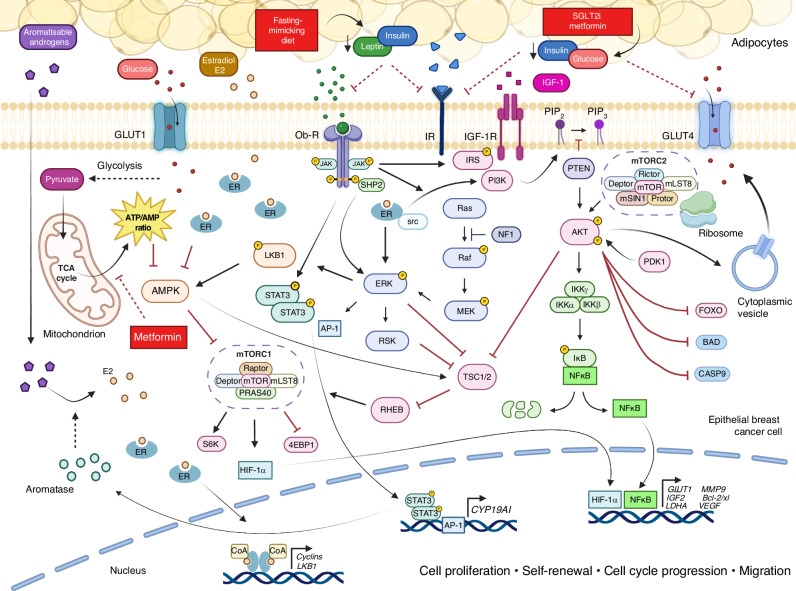
Insulin-PI3K–AKT–-mTOR signalling and the oestrogen receptor. The cellular energy landscape in breast cancer is regulated by a complex network of metabolic and signalling pathways. The PI3K/AKT signalling cascade, triggered by insulin and IGF-1, leads to mTORC1 activation driving protein synthesis and cancer progression. There is cross-talk between the PI3K–AKT–mTOR pathway and oestrogen receptor (ER) signalling at multiple levels and several preclinical studies have shown that the PI3K–AKT–mTOR pathway plays a key role in mediating resistance to endocrine therapy in breast cancer. Dietary and drug interventions such as fasting-mimicking diets and SGLT2 inhibitors or metformin reduce circulating insulin levels, offering potential therapeutic value in combination with endocrine therapy. Inflammatory signalling via the NFκB and JAK/STAT pathways also contributes to the regulation of genes that are crucial for cell survival and growth. The MAPK/ERK pathway, activated by insulin and leptin, has cross-talk with the PI3K/AKT pathway mediating activity of several downstream targets that collectively promote cell proliferation and cell-cycle progression. AMPK, the key regulator of energy homoeostasis in the cell, is inactivated in the presence of high ATP levels, which are influenced by increased glucose uptake and reliance on aerobic glycolysis, especially under hypoxic conditions. The antidiabetic drug metformin can indirectly activate AMPK, the key regulator of energy homoeostasis in the cell, by inhibiting mitochondrial complex I, leading to a shift in cellular energy balance, which in turn may contribute to the inhibition of mTOR activity. GLUT1 glucose transporter 1, Ob-R leptin receptor, JAK Janus kinase, SHP2 src homology 2-containing protein tyrosine phosphatase-2, ER oestrogen receptor, IR insulin receptor, IGF-1R insulin-like growth factor 1 receptor, IRS insulin receptor substrate, PI3K phosphoinositide 3-kinase, PIP_2_ phosphatidylinositol 4,5-bisphosphate, PIP_3_ phosphatidylinositol 3,4,5-trisphosphate, PTEN phosphatase and tensin homologue, AKT protein kinase B, mTORC1 mTOR complex 1, Raptor regulatory-associated protein of mTOR, Deptor DEP domain-containing mTOR-interacting protein, PRAS40 proline-rich Akt substrate of 40 kDa, mTORC2 mTOR complex 2, Rictor rapamycin-insensitive companion of mTOR, mSIN1 mammalian stress activated MAP kinase-interacting protein 1, Protor protein observed with Rictor-1, PDK1 phosphoinositide-dependent kinase-1, FOXO forkhead box O, BAD Bcl-2-associated death promoter, CASP9 caspase-9, IKKα I kappa B kinase alpha, IKKβ I kappa B kinase beta, IKKγ I kappa B kinase gamma, Iκb inhibitor of kappa B, NFκB nuclear factor kappa B, TSC1/2 tuberous sclerosis complex 1/2, RHEB Ras homologue enriched in brain, ERK extracellular signal-regulated kinase, MEK mitogen-activated protein kinase kinase, RSK ribosomal S6 kinase, AP-1 activator protein 1, STAT3 signal transducer and activator of transcription 3, LBK1 liver kinase B1, AMP adenosine monophosphate, ATP adenosine triphosphate, AMPK AMP-activated protein kinase, S6K S6 kinase, 4E-BP1 eukaryotic translation initiation factor 4E-binding protein 1, HIF-1α hypoxia-inducible factor 1-alpha, CYP19A1 cytochrome p450 family 19 subfamily A member 1, GLUT4 glucose transporter 4, LDHA lactate dehydrogenase A, MMP9 matrix metallopeptidase 9, VEGF vascular endothelial growth factor. (Created with BioRender.com.).

PI3K–AKT–mTOR is a key signal transduction pathway that mediates cell growth, metabolism, and cell survival. PI3K–AKT–mTOR integrates upstream signals, including those from insulin and insulin growth factors (IGF-1 and IGF2) as well as cellular nutrients, energy and oxygen levels (Fig. 2). There is cross-talk between the PI3K–AKT–mTOR pathway and the oestrogen receptor (ER) pathway at multiple levels [87, 88].

Genetic alterations affecting different nodes of the PI3K–AKT–mTOR pathway are common in ER+ breast cancer [89]. The international data sharing consortium, AACR Project GENIE, showed that genetic alterations in *PIK3CA*, *PTEN* and *AKT1* occur in ~36%, 7% and 5% of breast cancer, respectively [90]. It is not known whether these activating mutations are more likely to arise in breast cancer for patients that have insulin resistance or may influence the response to fasting in the context of ET.

Activation of the insulin receptor (IR) promotes downstream PI3K–AKT–mTOR signalling (see Fig. 2). Increased insulin levels are associated with higher breast cancer incidence and mortality [91]. Studies with both insulin analogues, blocking and stimulating anti-IR antibodies, and small molecule inhibitors, have shown a role for insulin signalling in breast cancer development and progression [92]. IR expression in breast cancer is well described, and high IR expression has been implicated in poor prognosis [93]. IR is more commonly expressed in endocrine-resistant breast cancer, and low expression correlates with improved survival [94].

Several preclinical studies have shown that the PI3K–AKT–mTOR pathway plays a key role in mediating resistance to ET in breast cancer, and the concept of targeting the PI3K–AKT–mTOR pathway to augment ET has now been proven in clinical settings. The BOLERO-2 Phase 3 trial demonstrated an improvement in median progression-free survival when the mTOR inhibitor everolimus was combined with the aromatase inhibitor exemestane in patients already refractory to single-agent aromatase inhibitor therapy [95]. The SOLAR-1 Phase 3 study has now also shown that the combination of the PI3K inhibitor, alpelisib, with fulvestrant led to an improvement in progression-free survival versus fulvestrant alone in PIK3CA-mutant, ER+ metastatic breast cancer resistant to first-line ET [96]. The Phase 3 placebo-controlled CAPltello-291 trial reported an improvement in progression-free survival with the addition of the AKT inhibitor, capivasertib to fulvestrant in patients with ER+ advanced breast cancer, irrespective of PIK3CA mutation status [97]. Lastly, the combination of inavolisib (a novel PI3K inhibitor) + palbociclib + fulvestrant in ER+ve metastatic breast cancer showed a significant improvement in investigator-assessed progression-free survival [98].

Drugs that lower circulating glucose and insulin levels, in particular metformin and SGLT2 inhibitors, have been proposed as treatments for breast cancer and could potentially synergise with ET by reducing PI3K–AKT–mTOR signalling (Fig. 2) [99, 100]. In particular, metformin has been extensively studied as a potential anticancer therapy, and a number of window-of-opportunity clinical trials have suggested that metformin may reduce cancer cell proliferation, and this effect may be greater in insulin-resistant women [100]. One meta-analysis of 11 observational studies has reported improved overall and cancer-specific survival in patients with breast cancer and diabetes who received metformin when compared with patients receiving other antidiabetic treatments [101]. However, another pharmacodynamic clinical study showed no clear link between metformin-induced reductions in circulating insulin levels and changes in tumour biology [102]. A large Phase 3 trial of 5 years of adjuvant metformin therapy in breast cancer showed no evidence of clinical benefit, although this study excluded patients with diabetes [103].

Aside from targeted therapies, dietary interventions have the potential to modulate the PI3K–AKT-mTOR pathway. Caffa et al. found that combining a periodic or fasting-mimicking diet (FMD) with hormone therapy, specifically fulvestrant and tamoxifen, enhanced anticancer effects in ER+ breast cancer mouse models by reducing leptin, insulin, and IGF-1 levels. Besides promoting sustained tumour regression, this approach could also revert acquired drug resistance [104].

#### Adipokine dysregulation and breast cancer

Investigating adipocyte biology, which goes beyond passive fat storage, is essential for comprehending the microenvironmental alterations linked to obesity. Adipocytes modulate the adipose tissue microenvironment through adipokine-mediated paracrine and autocrine signalling pathways. Two key adipokines involved in breast carcinogenesis are leptin and adiponectin.

Excess body fat increases leptin release from adipocytes, and BMI correlates with elevated leptin levels. By stimulating its receptor and a number of downstream pathways, including Jak2/Stat3, MAPK and PI3K–AKT, leptin likely promotes cell invasion and proliferation [104, 105]. A meta-analysis of 35 studies linked higher serum leptin levels with increased breast cancer risk, especially in postmenopausal women, suggesting its potential as a biomarker [106]. In addition, genetic variations in the leptin-coding genes *LEP* and *ADIPOQ* have been associated with elevated breast cancer risk [107]. Leptin has also been implicated in resistance mechanisms to tamoxifen and aromatase inhibitor treatment [108, 109].

Visceral adipose tissue (VAT) is known to produce leptin [50], but the effect of locally generated leptin from breast fat tissue compared to circulating leptin on breast tumour progression is not well understood.

On the other hand, low levels of adiponectin are associated with obesity and Type 2 diabetes, and studies suggest adiponectin may suppress cancer growth by modulating a number of intracellular metabolism and proliferation pathways associated with mitogenesis, including TNF-alpha, AMPK and SREBP-1 signalling [110, 111]. Unfavourable outcomes for breast cancer have been linked to both low adiponectin levels and increased leptin levels [112, 113], and it is speculated that the adiponectin:leptin ratio may be more important for breast cancer growth than the absolute levels [114].

Because high leptin levels are associated with an increased risk of breast cancer and may increase resistance to ET, as demonstrated in preclinical breast cancer models [115, 116], it has been speculated that lowering leptin levels through weight loss may improve outcomes for breast cancer survivors. Furthermore, a recent randomised study in this population demonstrated that both exercise and weight loss were associated with decreased leptin expression and improvements in the adipokine/leptin ratio [117], although whether this definitively translates to better clinical outcomes remains unanswered.

#### Obesity and FGF1, FGF2 and FGFR signalling

Another obesity-associated marker of elevated breast cancer risk, especially in the case of visceral fat, is fibroblast growth factor-2 (FGF2), which is released by adipose tissue. FGF2 binds to FGFR1 and FGFR2, and at least 10% of breast cancers harbour FGFR1 amplification, which is linked to early relapse and poor prognosis [118]. FGFR1 signalling directs healthy mammary duct development [119], and FGF2 levels are lowered in mice that have had a substantial fat pad removed, suggesting that FGF2 may have endocrine-mediated functions in addition to local ones [120]. Poor prognosis in breast cancer has been associated with elevated expression of the leptin receptor (LepR) and FGFR1 amplification, and co-expression of the FGFR1 gene and leptin protein copy number has been observed in primary breast tumours [121]. Antagonism of FGFR signalling in an obese mouse breast cancer model prevented outgrowth of pulmonary metastases [122] and FGFR inhibitors have already shown some promise in the clinic for the treatment of endocrine-resistant ER+ breast cancer [123].

In preclinical studies, elevated circulating levels of FGF2 have been linked to breast cancer development through the activation of oncogenic signalling pathways, including MAPK/ERK, cMYC and PI3K/AKT/mTOR. FGFR1 amplification is a key driver of ET resistance through MAPK signalling activation, and this therapeutic opportunity is currently being explored in clinical trials [124, 125]. Direct targeting of FGF2 is also being considered as a potential clinical approach [126].

FGF1 promotes adipocyte glucose uptake through AKT cross-talk as well as transcriptional promotion of glucose transporter protein type 1 (GLUT1), the primary glucose transporter [127–129]. GLUT1 is associated with higher grade, proliferation, as well as poorer prognosis in breast cancer [130, 131], although no link was observed between GLUT1 expression in breast cancer and background obesity or diabetes in one small study [132]. Notably, recent work has shown that FGF1 stimulates oestrogen receptor activation in obese mouse breast cancer models after oestrogen deprivation [128].

#### Aromatase overexpression in obesity

Aromatase inhibitors (AI) play a pivotal role in the treatment of ER+ breast cancer as a monotherapy in postmenopausal women. Postmenopausal status leads to a shift in the primary site of aromatase activity to the adipose tissue in the breast and gluteal areas. It is well described that AIs are less efficient at suppressing serum oestradiol levels in obese women [10]. A plausible explanation for this reduced efficiency is the observation that women with BMI >30, both with and without breast cancer, have elevated baseline oestrogen levels compared to those with BMI <22, and this may result in less effective suppression of oestrogen by an AI in postmenopausal women [133].

The formation of CLS in obesity is associated with heightened levels of gene transcription and increased activity of aromatase in mammary glands and visceral fat [51]. Aromatase expression is especially elevated in the adipose fibroblasts near breast tumours through the activation of proximal promoters, with immature fibroblasts primarily responsible for its production [134]. Furthermore, tumour cells in adipose tissue inhibit adipocyte differentiation by release of TNF-alpha and interleukin-11, thereby increasing the fibroblast:adipocyte ratio. This shift sustains elevated aromatase production, promoting local oestrogen synthesis and tumour progression [135].

A systematic review of three randomised controlled trials and five retrospective cohort studies suggested reduced efficacy of aromatase inhibitors in obesity, although the exact magnitude of this effect is not clearly established [136]. In a recent nationwide cohort study of 13,000 patients with hormone receptor-positive breast cancer, Harborg et al. showed that the risk of recurrence was higher among patients with obesity compared to those with a healthy weight (BMI 18.5–24.9) [4]. The ATAC study, which randomly assigned postmenopausal women with early-stage breast cancer to receive oral daily anastrozole alone, tamoxifen alone, or the combination, supported these findings. Specifically, women on anastrozole had a 27% lower recurrence rate compared to those taking tamoxifen, with women having a BMI <23 deriving an even greater benefit from treatment with an aromatase inhibitor [9].

In premenopausal women, AIs should be combined with ovarian suppression, typically with gonadotropin-releasing hormone (GnRh) analogues. Alternatively, selective oestrogen receptor modulators, such as tamoxifen, may be used alone or in combination with GnRh analogues. In premenopausal patients with HR-positive breast cancer who received adjuvant tamoxifen, a high BMI has been linked to a poorer prognosis [137]. However, to date, no similar association has been reported when ovarian suppression is used in conjunction with aromatase inhibitors.

AIs are usually provided at a standard dose that does not take specific inter-patient variation into consideration. Early studies investigating whether a larger dosage of AIs may improve outcomes for obese individuals with metastatic cancer suggested no additional benefits from an increased dose. However, these trials were conducted prior to the introduction of AIs as a standard-of-care option for postmenopausal ER+ breast cancer and therefore weight-dependent dosing may be revisited [138, 139].

Another issue demanding attention is the heightened insulin resistance and increased adiposity associated with postmenopausal women with breast cancer undergoing treatment with AIs. Studies in aromatase knockout mice and rare cases of congenital aromatase deficiency indicate a correlation with elevated adiposity, hepatic steatosis and insulin resistance [140–142]. Consequently, the advantages of ET must be weighed against the potential risks of obesity, metabolic syndrome and diabetes. This emphasizes the need for further investigation as to whether these changes in metabolism are associated with a worse prognosis and whether early drug or dietary intervention might be beneficial.

## Implications for toxicity

It has long been recognised that there is a challenge in correctly dosing obese patients with adjuvant chemotherapy, as the maximum tolerated dose for cytotoxic therapy will have typically been determined in a leaner population. Obese patients are thought to be often underdosed due to empirical dose reductions, contrary to guidelines recommending full weight-based dosing. Although there is limited clinical data, it is hypothesised that this may lead to worse outcomes [143, 144]. There is also considerable uncertainty when using drugs with a high risk of cumulative toxicity, such as doxorubicin, fluoropyrimidines and cyclophosphamide. Studies are ongoing to determine whether better measures of body composition can more accurately predict toxicity in early breast cancer treatment [145].

Furthermore, studies have shown that obese patients with breast cancer receiving ET experience increased adverse effects, such as increased joint symptoms and cardiovascular events, which could potentially lead to treatment discontinuation [146, 147]. Hence, lack of treatment compliance in this context may be a contributing factor to poorer outcomes. Notably, in the context of treatment with adjuvant CDK4/6 inhibitors, obese patients had lower rates of neutropaenia which translated into a reduced treatment discontinuation rate in the PALLAS trial. The investigators hypothesised that obese patients may have a lower distributional volume, although survival data are still immature [148]. This suggests a need for dosage adjustments based on body composition rather than standard weight-based protocols to maximise therapeutic effects in obese patients.

The use of mTOR and PI3K inhibitors in conjunction with ET for the treatment of breast cancer may be especially problematic in patients with obesity or insulin resistance. Using clinical trial data from two studies of PI3K inhibitors, Rodon et al. developed a risk prediction model for grade 3/4 hyperglycaemia, and identified five factors, including baseline fasting plasma glucose, HbA1c and BMI, as the strongest predictors for classifying patients as low or high risk [149]. Notably, preclinical research has shown that the insulin feedback causing hyperglycaemia can be prevented using dietary or pharmaceutical approaches, which greatly enhance the efficacy of treatment [150].

## Conclusion

For some time, it has been understood that obesity and insulin resistance are associated with both an increased risk of developing ER+ breast cancer and poorer outcomes. Substantial preclinical evaluation has now provided greater insight into the mechanisms that drive these phenomena, and potential therapeutic strategies have been proposed. Clinical studies of interventions aimed at improving outcomes for breast cancer patients with metabolic disorders are warranted. More accurate measures of body composition beyond BMI and their association with patient outcome need to be assessed in the clinic and potential differences in treatment resistance between premenopausal and postmenopausal women in the context of obesity remain understudied. Lastly, the breast cancer community needs to evaluate strategies to effectively manage treatment toxicity in the context of obesity.
